# Supplementary material for: Integrating single-cell and bulk transcriptomic perturbation resources reveals complementary therapeutic spaces for drug repurposing
Source: bioRxiv. 2026 Jul 22:2026.07.17.739227. Preprint. [Version 1] doi: 10.64898/2026.07.17.739227 (PMC13419695; doi:10.64898/2026.07.17.739227)
Supplement: Supplement 1 [file NIHPP2026.07.17.739227v1-supplement-1.pdf]

bioRxiv preprint doi: https://doi.org/10.64898/2026.07.17.739227; this version posted July 22, 2026. The copyright holder for this preprint (which was not certified by peer review) is the author/funder, who has granted bioRxiv a license to display the preprint in perpetuity. It is made available under a CC-BY 4.0 International license.

SUPPLEMENT:

Supplement Algorithm 1: Calculation of Connectivity Score

Algorithm 1 Calculation of Connectivity Scores (CS)

Require: Disease gene sets S_{up}, S_{down}; Drug Profile L; Universe size N.

Ensure: Raw Connectivity Score (CS).

procedure GETSCORE(S_{up}, S_{down}, L)
  Calculate KS_{up} for S_{up}:
  $a_{up} \leftarrow \max_{j=1}^{n_{up}} \left(\frac{j}{n_{up}} - \frac{\text{rank}(g_j)}{N} \right)$
  $b_{up} \leftarrow \max_{j=1}^{n_{up}} \left(\frac{\text{rank}(g_j)}{N} - \frac{j-1}{n_{up}} \right)$
  if $a_{up} > b_{up}$ **then** $KS_{up} \leftarrow a_{up}$
  else $KS_{up} \leftarrow -b_{up}$
  end if
  Calculate KS_{down} similarly for S_{down}.
  Compute Score (CS):
  if $\text{sign}(KS_{up}) \neq \text{sign}(KS_{down})$ **then**
    $CS \leftarrow KS_{up} - KS_{down}$
  else
    $CS \leftarrow 0$
  end if
  return CS
end procedure

Supplement Algorithm 2: Statistical Assessment and Candidate Selection

bioRxiv preprint doi: https://doi.org/10.64898/2026.07.17.739227; this version posted July 22, 2026. The copyright holder for this preprint (which was not certified by peer review) is the author/funder, who has granted bioRxiv a license to display the preprint in perpetuity. It is made available under a CC-BY 4.0 International license.

Algorithm 2 Statistical Assessment and Candidate Selection

Require: Drug Database $\mathcal{D}$; Permutations $M = 100,000$; FDR $\alpha = 0.05$.

Ensure: List of significant reversal drugs $\mathcal{R}$.

procedure ASSESSSIGNIFICANCE($\mathcal{D}$)
  Initialize observed scores list $\mathcal{S}_{obs} \leftarrow []$.
  ▷ Step 1: Calculate Observed Scores
  for each drug $L \in \mathcal{D}$ **do**
    $CS_{obs} \leftarrow$ GETSCORE($\mathcal{S}_{up}, \mathcal{S}_{down}, L$) ▷ Calls Algorithm 1
    Append CS_{obs} to $\mathcal{S}_{obs}$.
  end for
  ▷ Step 2: Generate Null Distribution
  Generate M random drug profiles.
  Calculate CS_{null} for all random profiles.
  ▷ Step 3: Calculate P-values (Phipson & Smyth)
  for each $CS_{obs} \in \mathcal{S}_{obs}$ **do**
    $b \leftarrow \sum_{k=1}^{M} \mathbb{I}(|CS_{null}^{(k)}| \geq |CS_{obs}|)$
    $p_{val} \leftarrow \frac{b+1}{M+1}$
  end for
  Compute q-values (Benjamini-Hochberg).
  $\mathcal{R} \leftarrow \{\text{drugs} \mid CS < 0 \text{ and } q < \alpha\}$
  return $\mathcal{R}$
end procedure

Supplement Fig 1: CDRPipe Shiny application workflows

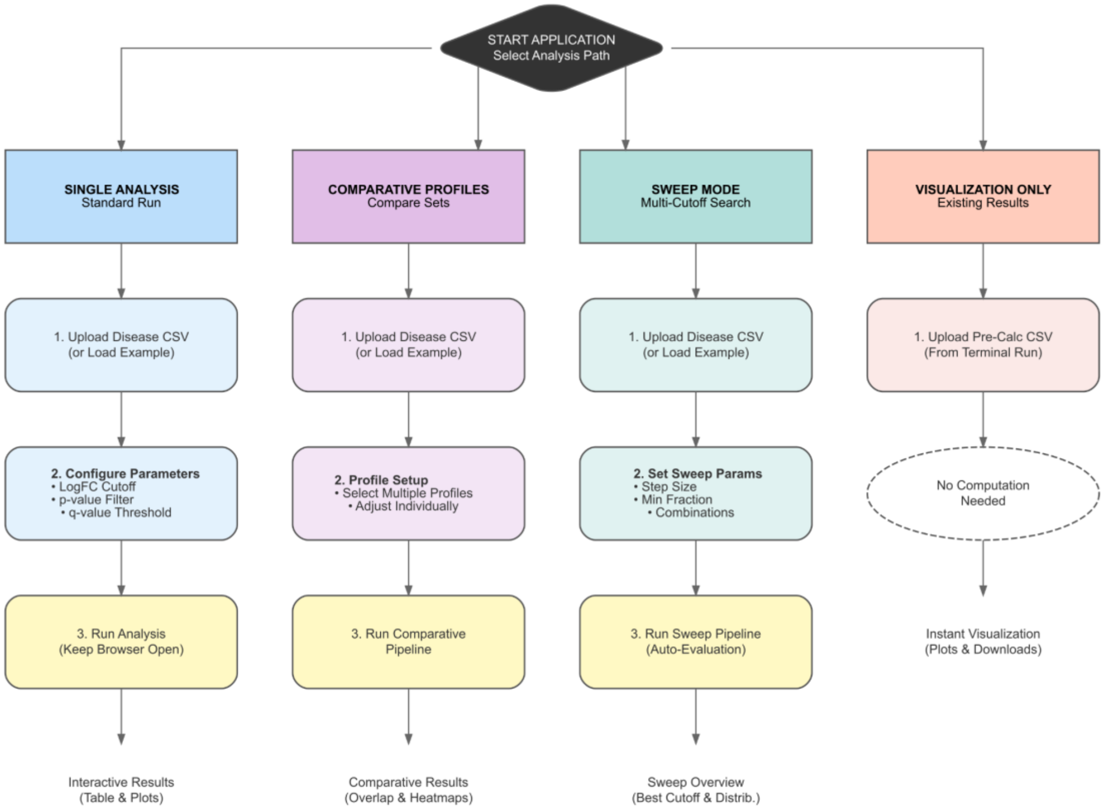

bioRxiv preprint doi: https://doi.org/10.64898/2026.07.17.739227; this version posted July 22, 2026. The copyright holder for this preprint (which was not certified by peer review) is the author/funder, who has granted bioRxiv a license to display the preprint in perpetuity. It is made available under a CC-BY 4.0 International license.

Supplement Fig. 1. CDRPipe Shiny Application Workflows. The application interface guides users through four distinct analysis modules starting from the main dashboard: **(1) Single Analysis:** A standard workflow for running drug repurposing on individual disease signatures. **(2) Comparative Profiles:** A module for analyzing and contrasting multiple disease sets simultaneously to identify shared or distinct therapeutic signals. **(3) Sweep Mode:** An optimization tool that performs a multi-cutoff grid search to identify ideal parameter thresholds. **(4) Visualization Only:** A rapid-access mode for rendering plots from pre-computed results files without re-running the computational pipeline. Each path follows a structured workflow of data upload, parameter configuration, execution, and interactive result exploration.

Supplement Fig 2: CDRPipe R Shiny App Home Page

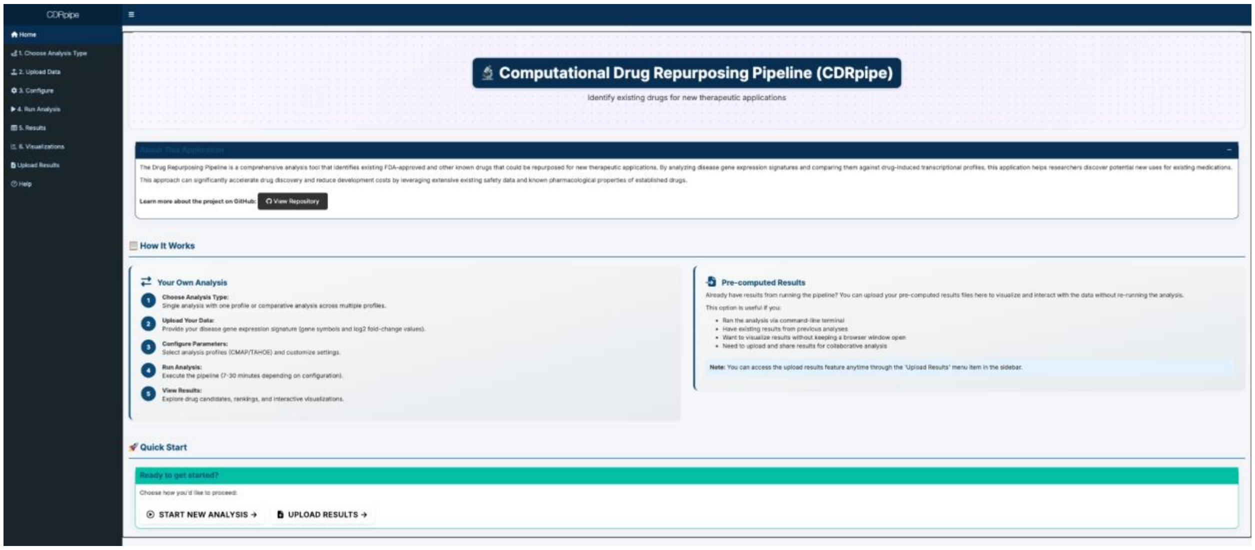

Supplement Fig. 2. CDRPipe R Shiny Application Home Page. The figure displays the landing interface of the web application, which serves as the central hub for the DRPipe workflow. The dashboard features a **"How It Works"** section that outlines the step-by-step process for conducting a new analysis, from choosing an analysis mode and uploading gene expression signatures to configuring parameters and interpreting results. Additionally, the interface offers a **"Pre-computed Results"** pathway, allowing users to upload and visualize existing pipeline outputs without re-executing the computational steps. Navigation is facilitated through the sidebar menu and **"Quick Start"** action buttons.

Supplement Fig 3: Schematic overview of the CDRPipe workflow

bioRxiv preprint doi: https://doi.org/10.64898/2026.07.17.739227; this version posted July 22, 2026. The copyright holder for this preprint (which was not certified by peer review) is the author/funder, who has granted bioRxiv a license to display the preprint in perpetuity. It is made available under a CC-BY 4.0 International license.

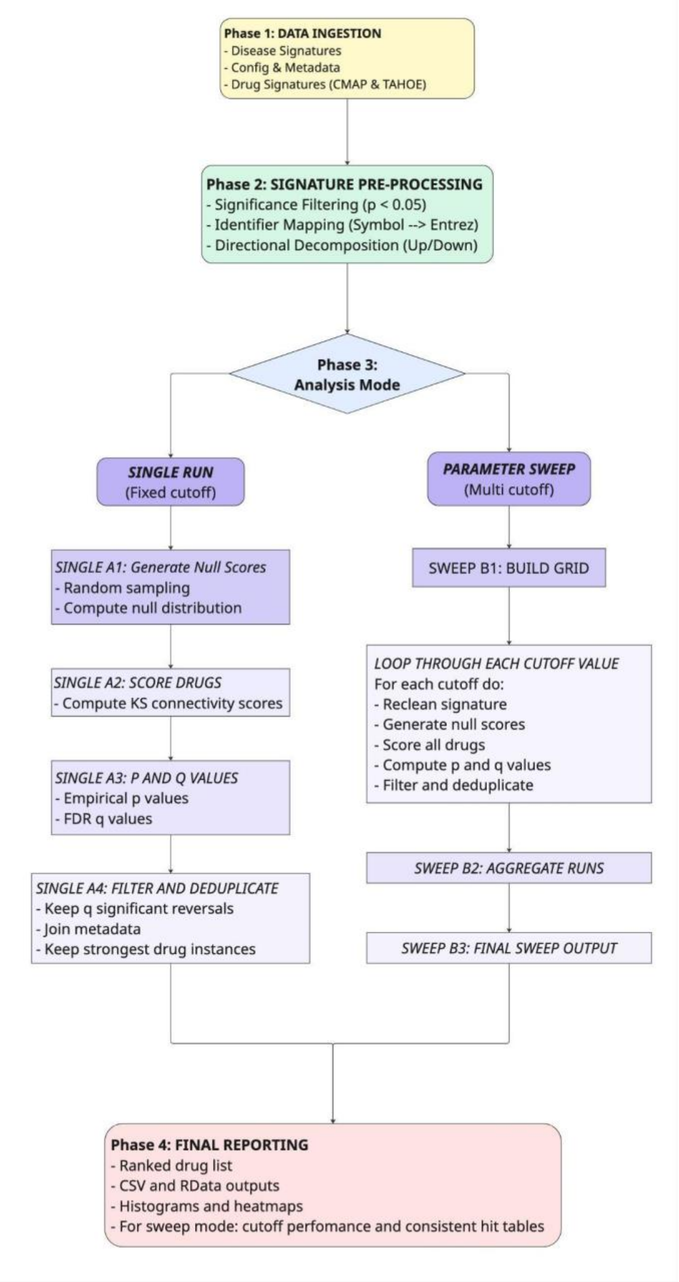

Supplement Fig. 3. Schematic overview of the DRPipe workflow. The pipeline proceeds in four distinct phases: **(1) Data Ingestion** of disease signatures and drug perturbation profiles (CMAP and Tahoe-100M). **(2) Signature Pre-processing**, which standardizes inputs via significance filtering ($p < 0.05$), identifier mapping to Entrez IDs, and directional decomposition into up/down gene sets. **(3) Analysis Mode**, where users select between a **Single Run** (using fixed cutoffs) or a **Parameter Sweep** (optimizing thresholds via grid search). This phase executes the core algorithm: generating null distributions, computing Kolmogorov-Smirnov (KS) connectivity scores, and assessing statistical significance via empirical p-values and FDR correction. **(4) Final Reporting**, which outputs ranked candidate lists, visualization plots, and comprehensive result tables for downstream validation.

bioRxiv preprint doi: https://doi.org/10.64898/2026.07.17.739227; this version posted July 22, 2026. The copyright holder for this preprint (which was not certified by peer review) is the author/funder, who has granted bioRxiv a license to display the preprint in perpetuity. It is made available under a CC-BY 4.0 International license.

Supplement Table 1: Autoimmune Disease Summary

Disease	Known Drugs (DB)	Available (CMAP)	Hits (CMAP)	Recovered (CMAP)	Recovery Rate (CMAP)	Available (TAHOE)	Hits (TAHOE)	Recovered (TAHOE)	Recovery Rate (TAHOE)	Total Candidates	Total Recovered	Overall Recovery Rate
Scleroderma	18	4	300	2	50.00%	1	230	1	100.00%	978	3	16.67%
Autoimmune thrombocytopenic purpura	53	16	28	2	12.50%	11	379	11	100.00%	474	13	24.53%
Psoriasis	149	20	273	15	75.00%	14	332	14	100.00%	574	25	16.78%
Psoriatic arthritis	47	9	5	0	0.00%	6	365	6	100.00%	709	6	12.77%
Inclusion body myositis	8	1	7	0	0.00%	1	357	1	100.00%	419	1	12.50%
Discoid lupus erythematosus	9	1	6	0	0.00%	2	357	2	100.00%	433	2	22.22%
Dermatomyositis	29	4	93	0	0.00%	2	370	2	100.00%	463	2	6.90%
Sjogren's syndrome	39	6	701	4	66.67%	4	315	4	100.00%	1086	8	20.51%
Inflammatory bowel disease	33	11	109	1	9.09%	7	345	6	85.71%	503	6	18.18%
Ankylosing spondylitis	43	12	7	0	0.00%	9	272	7	77.78%	417	7	16.28%
Crohn's disease	104	27	102	2	7.41%	12	332	9	75.00%	476	11	10.58%
Relapsing-remitting multiple sclerosis	79	19	679	6	31.58%	9	248	6	66.67%	1065	12	15.19%
Rheumatoid arthritis	240	45	164	8	17.78%	25	272	16	64.00%	859	22	9.17%
Systemic lupus erythematosus	103	20	268	8	40.00%	15	267	9	60.00%	894	16	15.53%
Ulcerative colitis	115	24	68	3	12.50%	10	207	5	50.00%	619	8	6.96%
Multiple sclerosis	145	43	247	12	27.91%	16	141	7	43.75%	774	18	12.41%
Juvenile idiopathic arthritis (sJIA)	10	11	72	0	0.00%	8	81	3	37.50%	191	3	30.00%
Type 1 diabetes mellitus	138	31	201	8	25.81%	12	178	2	16.67%	807	10	7.25%

Supplement Table 1. Comparative Performance Metrics Across 18 Autoimmune Diseases. The table provides a detailed breakdown of drug recovery statistics for each disease analyzed in Case Study 1. Columns quantify the Known Drugs (total validated treatments in Open Targets), the subset Available within each platform's library, the total number of significant predictions (Hits), and the number of validated predictions (Recovered) for both CMAP and Tahoe-100M. The Recovery Rate columns highlight the performance disparity between the platforms; notably, Tahoe-100M achieves 100% recovery of available known drugs for 8 of the 18 conditions (e.g., Psoriatic Arthritis, Inclusion Body Myositis), whereas CMAP frequently shows 0% recovery for the same indications.

bioRxiv preprint doi: https://doi.org/10.64898/2026.07.17.739227; this version posted July 22, 2026. The copyright holder for this preprint (which was not certified by peer review) is the author/funder, who has granted bioRxiv a license to display the preprint in perpetuity. It is made available under a CC-BY 4.0 International license.

Supplement Table 2: CMap Drugs List:
https://drive.google.com/file/d/1-_eaCW600Tu7tE-cDOyqqC1Xn8KUprof/view?usp=sharing

Supplement Table 3: Tahoe-100M Drugs List:

https://drive.google.com/file/d/1TsjJeYhW-5p_BiENejMCv2EWiSPww0Fd/view?usp=sharing

Supplement Table 4: Tahoe-100M & CMap Drug Overlap List:

https://drive.google.com/file/d/13lZhEOnQF0sCBLTutsASkUW-C8lg9Z_T/view?usp=sharing

Supplement Table 4: CREEDs Disease Names List:

https://drive.google.com/file/d/1-hL2SkOfaylUiotNCU73zlv9bkxMAq0q/view?usp=sharing
